# Chronic pain in older adults with chronic diseases: prevalence, perceived interference, and management strategies

**DOI:** 10.1186/s12877-025-06823-7

**Published:** 2025-11-28

**Authors:** Pasiri Singhasiri, Chaisiri Angkurawaranon, Suphawita Pliannuom, Nopakoon Nantsupawat, Kanokporn Pinyopornpanish

**Affiliations:** 1 Department of Family Medicine, Faculty of Medicine, Chiang Mai University, 110 Inthawarorot Rd., Sriphum, Muang, Chiang Mai, 50200 Thailand; 2 Global Health and Chronic Conditions Research Group, Chiang Mai University, Chiang Mai, 50200 Thailand

**Keywords:** Chronic pain, Older adults, Pain prevalence, Pain interference

## Abstract

**Background:**

Chronic pain is a worldwide health concern in aging populations, often accompanying chronic diseases. Despite its prevalence, the impact of chronic pain in older adults is frequently underestimated. This study examines the prevalence, characteristics, and impact of chronic pain among older adults with chronic diseases.

**Methods:**

Cross-sectional data from 160 participants aged over 60 years with chronic diseases were collected, including their sociodemographic details, current pain status, and pain management. Data was analyzed using Fisher’s exact tests, Student’s t-test and multivariable linear regression to examine the relationships between pain interference and multiple variables.

**Results:**

The prevalence of current chronic pain was 63.75%, with 69.61% experiencing nociceptive pain. Half of the participants had not received assessment and diagnosis of the chronic pain from health care professionals. Significant associations were observed between perceived pain interference and age (Coefficient = 0.42; 95% CI: 0.07–0.76; p 0.017), maximum pain severity (Coefficient = 1.81; 95% CI: 0.86–2.76; *p* < 0.001), and pain frequency (Coefficient = 0.22; 95% CI: 0.04–0.39; p 0.015). Common pain management approaches included consultation of healthcare practitioners (40.20%) and alternative medicine (33.33%), with participants reporting high pain interference being more likely to seek practitioner care (58.33%) compared to those with low interference (24.07%, *p* = 0.001).

**Conclusion:**

The high prevalence of chronic pain and pain underestimation in older adults underscores the necessity for comprehensive pain management strategies adopting a patient-centered medicine approach. The provision of education to both physicians and patients on appropriate chronic pain management strategies and options may benefit older adults with chronic diseases.

## Background

Non-communicable diseases (NCDs) contribute significantly to the global disease burden, with aging populations experiencing a high prevalence of chronic diseases and chronic pain [1, 2]. Conditions such as musculoskeletal disorders, cardiovascular disease, diabetes, and neurological disorders frequently coexist, increasing the risk of functional decline, psychological distress, and diminished quality of life [3–6]. Chronic pain is defined as pain occurring on most days or every day over the past three months [7, 8]. It is a major health burden, affects daily activities, emotional well-being, and social interactions, further complicating the management of chronic diseases [9–13]. These findings emphasize the importance of the acknowledgement of the consequences of chronic diseases in the aging populations especially when the world’s population is aging rapidly [14].

Fredheim et al. [15] investigated the quality of life among chronic pain patients in Norway and found that individuals with chronic non-malignant pain often experience a lower quality of life compared to those with terminal cancer. Various studies have identified key risk factors for pain [13, 16–18], which include: (1) Sociodemographic factors: female gender, advanced age, lower socioeconomic status, lower education level; (2) Physical health conditions including chronic diseases such as diabetes, hypertension, obesity, stroke, urogenital disorders, chronic gastritis, hyperuricemia/gout, neuropathies, cardiac sufficiency, depression, interstitial lung disease, bone and spinal problems; and (3) Health risk behavior specifically, poor levels of physical activity and smoking. The impact of chronic pain is found in various dimensions in daily lives including daily activities, emotions, work, and interpersonal relationships [19–23] which are responsible for limitations in physical function and deterioration of quality of life [6, 24]. Consequently, inadequate pain management can result in a major decline in the health status of the older adults in addition to their chronic diseases [25]. There are notable risks associated with analgesics use in older adults, with some classified as potentially inappropriate medications (PIMs) due to increased risks of gastrointestinal bleeding, renal impairment, delirium, falls, and fractures - particularly with non-steroidal anti-inflammatory drugs (NSAIDs), opioids and muscle relaxants - highlighting the need for careful medication selection in these population [26].

Although chronic pain in older adults has a major impact, it is often overlooked by medical physicians especially in comparison to the patient’s own perceptions. This mismatched underestimation is likely to increase in line with pain severity and results in undertreatment of chronic pain [27]. Therefore, it is essential to understand the pain profiles and the significant impact of pain in the older adult population with chronic diseases to overcome this issue. In settings involving community-dwelling older adults in Asia, over one-third of older adult population in Thailand had experienced moderate to severe pain [17]; over 60% of older adults in Japan had experienced chronic pain, and a study in China showed that prevalence of chronic pain in older people with chronic diseases was over 90% [18]. However, most existing studies focused on pain location, and intensity, with limited exploration of how pain affects daily life or assessment of pain management strategies. In Thailand, the accessibility of pain management across different healthcare systems also remains under-documented. Furthermore, the reliance on non-pharmacological approaches (e.g., massage, cupping, needling, distraction, endurance) and the limited use of pharmacological self-management contrast with findings from cohorts in other countries, highlighting the influence of cultural attitudes and accessibility of pain medications. Thus, investigation into the type and management of chronic pain is crucial for the achievement of proper care and evaluation either by oneself or practitioners [17]. This study aims to investigate the prevalence, characteristics, perceived life interference, and management of chronic pain among older adults with chronic diseases in a Northern Thailand primary care setting. Findings from this research could help highlight the importance of comprehensive pain assessment, promote timely intervention, and encourage effective pain management strategies.

## Materials and methods

### Study design and setting

This cross-sectional study was conducted at the outpatient clinic (OPD) of the Department of Family Medicine, Maharaj Nakorn Chiang Mai Hospital, located in Chiang Mai province, Northern Thailand. The clinic primarily provides general health services and regular follow-up for adults and older adults with chronic conditions, particularly non-communicable diseases (NCDs). Data collection was carried out between April and July 2024.

As part of a university-affiliated tertiary care hospital, this clinic primarily serves patients under the Civil Servant Medical Benefit Scheme, which offers broader access to a wide range of medications compared to other healthcare coverage types in Thailand. Medical benefit status in Thailand is divided into three schemes: Civil Servant Medical Benefit Scheme (CSMBS), Social Security Scheme (SSS), and Universal Coverage Scheme (UC). CSMBS covers government employees, retirees, and their dependents, whereas SSS covers private-sector employees and UC covers the remainder of the population.

The pain management services provided in the clinic primarily include health education, lifestyle modification, and medication prescription. Referral to specialty services, such as pain or rehabilitation clinics, is available when pain can not be adequately managed by family physicians, depending on the patient’s condition. Patients can access self-medication practices and alternative treatments for pain, such as acupuncture, massage, or alternative medicines, which are available in hospitals, the private sector, or the community.

The sample size was calculated using the infinite population proportion formula. Based on previous research reporting a chronic pain prevalence of 59.86% among older adults with chronic diseases [18], a minimum of 145 participants was required to achieve 80% statistical power at a significance level of 0.05. To account for potential 10% incomplete responses, the final target sample size was set at 160 participants.

### Data collection

Eligible participants were initially screened by nurses at the OPD. A trained researcher assistant then obtained informed consent individually. Once participants agreed to take part, they were recruited and asked to complete the questionnaire with assistance if needed. The research assistant was not involved in any aspect of clinical care or treatment.

Information was gathered in three main areas:

1. Sociodemographic data: Age, gender, education level, occupation, medical benefits, place of residence, and household status (living alone, or with others).
2. Pain-related information: Previous history of pain diagnosis and assessment [we asked, ‘Have you previously received a pain assessment from any physician for your reported chronic pain?’ Participants who reported ‘yes’ were categorized into the “Assessed by physician” group, while those who answered ‘no’ were classified as the “Never been assessed.”], presence of current chronic pain [defined as persistent pain lasting for more than 3 months, beyond the normal healing time [8], and continuing up to the past 7 days], and pain-related details [pain-related details included pain descriptors, severity, frequency, and interference.] Pain descriptors were selected from predefined classifications based on the most predominant sensation reported by participants. These were categorized as follows: Nociceptive pain: dull pain, tightness, heaviness, fullness, throbbing, cramping, stabbing, splitting, piercing pain; Neuropathic pain: tingling, electric shock-like pain, burning pain, numbness/thickness/reduced sensation, pain upon light touch or wind, cold pain (like being touched by ice), and itching. We assessed pain frequency by asking participants to report how often they experienced pain during the past week. Pain severity and interference were assessed using the 11-item self-reported Brief Pain Inventory (BPI) scale [28]. Pain severity was rated on a numerical scale from 0 (no pain) to 10 (pain as bad as one can imagine). Pain interference was assessed based on how pain affected the following dimensions: general activity, mood, walking ability, normal work, relationships with others, sleep, and enjoyment of life. Each item was rated on a scale from 0 (does not interfere) to 10 (completely interferes). The total pain interference score was calculated by summing all seven BPI interference items. Patients were categorized as having high or low pain interference based on the median value of the total interference score.
3. Chronic pain management: Participants were asked “What method have you chosen to manage this chronic pain?” Responses were pre-categorized into common approaches, including enduring pain, using distraction techniques, self-seeking oral medication, consulting practitioners, and using alternative medicine practices [18, 29, 30]. To assess medication use, participants were also asked, “What medication have you used to manage your pain?” Information was collected on both self-medicated drugs and those prescribed by physician. Potentially Inappropriate Medications (PIMs) were defined according to the Beers Criteria [26] and included NSAIDs, opioids, benzodiazepines, antiepileptics, antipsychotics, and antidepressants. The use of alternative medicine practices was assessed through an open-ended question, allowing participants to freely describe their methods; however, responses were limited to practices provided by trained professionals.

### Data analysis

Categorical data is presented as percentages and was analyzed using the Fisher’s exact test, while continuous data are presented as mean values with standard deviation and analyzed using a Student’s t-test. For participants reporting current chronic pain, potential associations between various factors and perceived pain interference were examined using multivariable linear regression analysis. All analyses were conducted using Stata version 16, with statistical significance set at a p-value < 0.05. Results are reported with 95% confidence intervals.

## Results

**Fig. 1:**
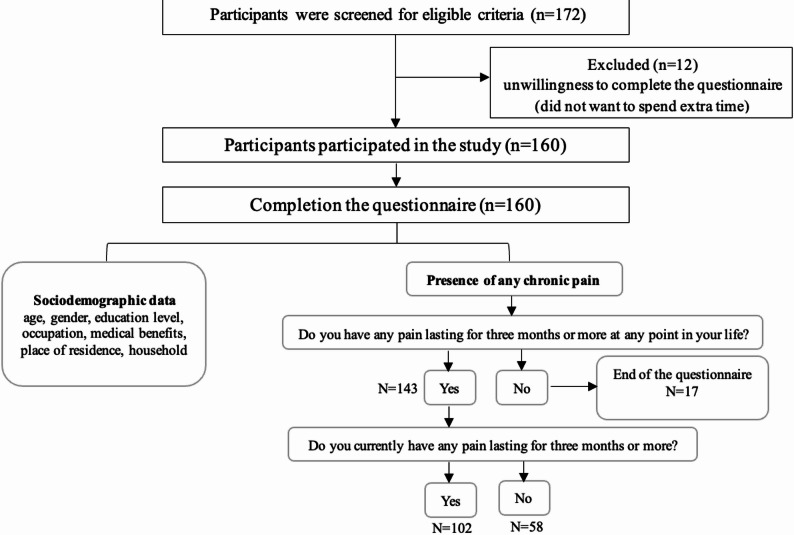
Flow diagram of the study

Out of 172 invited patients with NCDs, 160 provided informed consent to participate in the study, as shown in Fig. 1. Demographic data of participants are described in Table 1. Among the 160 participants, over two-thirds of participants reported currently experiencing chronic pain (102 patients, 63.75%). Based on the study’s definition of chronic pain (pain occurring on most days or every day over the past three months), 17 participants (10.62%) out of 160 had never experienced chronic pain, while 143 participants (89.38%) reported having experienced it at some point in their lives. Interestingly, among those with current chronic pain, half of the participants had not received assessment and diagnosis of the chronic pain from physicians.

**Table 1:** Sociodemographic data of participants

Characteristics	Total (*N* = 160)	Currently experiencing chronic pain (*N* = 102)		No current chronic pain (*N* = 58)		*p*-value
Female, *n* (%)	108(67.50)	73(71.57)		35(60.34)		0.163
Male, *n* (%)	52(32.50)	29(28.43)		23(39.66)		
Mean age, years ± SD	72.03 ± 6.33	71.47 ± 6.16		73.00 ± 6.55		0.142
Highest education, *n* (%)						
Below primary school	58(36.25)	38(37.25)		20(34.48)		0.864
Higer than primary school	102(63.75)	64(62.75)		38(65.52)		
Occupation, *n* (%)						
Retired	80(50)	48(47.06)		32(55.17)		0.330
Homemaker	60(37.50)	40(39.22)		20(34.48)		
Merchant	12(7.50)	10(9.80)		2(3.45)		
Employee	8(5)	4(3.92)		4(6.90)		
Medical benefit, *n* (%)						
Social Health Insurance	3(1.88)	1(0.98)		2(3.45)		0.436
Universal Coverage Scheme	2(1.25)	1(0.98)		1(1.72)		
Civil servant medical benefit	145(90.62)	92(90.20)		53(91.38)		
Pay by oneself	10(6.25)	8(7.84)		2(3.45)		
Living alone, *n* (%)						
Yes	144(90)	93(91.18)		51(87.93)		0.587
No	16(10)	9(8.82)		7(12.07)		
Residence location, *n* (%)						
Urban area	54(33.75)	34(33.33)		20(34.48)		1.000
Rural area	106(66.25)	68(66.67)		38(65.52)		
Chronic diseases,* n* (%)						
Dyslipidemia	156(97.50)	100(64.10)		56(35.90)		0.621
Hypertension	141(88.12)	87(61.70)		54(38.30)		0.204
Musculoskeletal	100(62.50)	68(68.00)		32(32.00)		0.175
High blood sugar	77(48.12)	47(61.04)		30(38.96)		0.514
Eye problems	54(33.75)	32(59.26)		22(40.74)		0.487
Genitourinary	49(30.62)	33(67.35)		16(32.65)		0.595
Gastrointestinal	38(23.75)	24(63.16)		14(36.84)		1.000
Respiratory	18(11.25)	12(66.67)		6(33.33)		1.000
Neurological	11(6.88)	7(63.64)		4(36.36)		1.000
Cardiovascular	9(5.72)	6(66.67)		3(33.33)		1.000
Psychological	9(5.62)	5(55.56)		4(44.44)		0.724
Mean chronic diseases	4.40 ± 1.32	4.39 ± 1.44		4.41 ± 1.09		0.921
Cause of chronic pain, *n* (%)						
Assessed by physician	-	51 (50.00)		-		
Never been assessed	-	51 (50.00)		-		

Of the 102 participants currently experiencing chronic pain, nociceptive pain was the most prevalent, accounting for 69.61% of cases, more than twice the prevalence of neuropathic pain (30.39%). The most reported pain locations were the lower extremities (44.12%), followed by the back (22.55%) and the neck/shoulder (13.73%). As shown in Table 2, among participants with currently experiencing chronic pain: pain intensity scores were significantly higher in the high interference group than in the low interference group (*p* < 0.001). Pain interference across all life domains (daily activities, leisure, mobility, sleep, work, mood, and relationships) was significantly greater in the high interference group (all *p* < 0.05). The most affected domains were mobility (mean = 4.29) and daily activities (mean = 3.79) in the high interference group.

**Table 2:** Pain descriptors, sites, and intensity among participants with chronic pain categorized by perceived interference (*N* = 102)

	Total	Interference		*p*-value
	*N* = 102	Low *N* = 54	High *N* = 48	
Pain descriptors, *n* (%)				0.832
Nociceptive	71(69.61)	37(68.52)	34(70.83)	
Neuropathic	31(30.39)	17(31.48)	14(29.17)	
Pain location, *n* (%)				0.090
Face/Head	1(0.98)	0(0)	1(2.08)	
Neck/Shoulder	14(13.73)	9(16.67)	5(10.42)	
Back	23(22.55)	8(14.81)	15(31.25)	
Arm/Hand	13(12.75)	5(9.26)	8(16.67)	
Abdomen	1(0.98)	0(0)	1(2.08)	
Hip	5(4.90)	4(7.41)	1(2.08)	
Leg/Knee/Foot	45(44.12)	28(51.85)	17(35.42)	
Pain intensity, Mean ± SD				
Pain severity				
Worst pain	6.11 ± 2.51	5.24 ± 2.59	7.10 ± 2.01	< 0.001
Average pain	3.55 ± 1.74	2.76 ± 1.45	4.44 ± 1.61	< 0.001
Least pain	2.28 ± 1.55	1.61 ± 1.03	3.04 ± 1.69	< 0.001
Current pain	1.40 ± 1.94	0.5 ± 0.88	2.42 ± 2.29	< 0.001
Pain interference domain				
Daily activities	2.29 ± 2.34	0.96 ± 1.19	3.79 ± 2.41	< 0.001
Leisure activities	1.33 ± 1.99	0.31 ± 0.64	2.48 ± 2.35	< 0.001
Mobility	2.73 ± 2.85	1.33 ± 1.60	4.29 ± 3.13	< 0.001
Sleeping	1.81 ± 2.57	0.65 ± 1.23	3.12 ± 3.03	< 0.001
Working	1.72 ± 2.38	0.42 ± 0.86	3.17 ± 2.71	< 0.001
Mood	1.18 ± 2.35	0.20 ± 0.66	2.27 ± 3.02	< 0.001
Relationship	0.25 ± 1.06	0	0.54 ± 1.50	0.005

A multivariable linear regression analysis was used to examine the relationship between chronic pain interference and various factors. The findings revealed significant positive associations between chronic pain interference and the following variables: age (Coefficient = 0.42; 95% CI: 0.07, 0.76; p 0.017), maximum pain severity (Coefficient = 1.81; 95% CI: 0.86, 2.76; *p* < 0.001), and pain frequency (Coefficient = 0.22; 95% CI: 0.04, 0.39; p 0.015), as detailed in Table 3.

**Table 3:** Factors associated with perceived interference from current chronic pain

Variables	Coefficient (*β*)	95% CI	*p*-value
Age (years)	0.42	0.07, 0.76	0.017
Female	-3.64	-8.05, 0.76	0.104
Living alone	-4.60	-11.73, 2.52	0.203
Urban area	1.31	-3.01, 5.63	0.548
Number of chronic diseases	-0.27	-1.72, 1.17	0.708
Assessed by physician	-1.17	-5.88, 3.54	0.624
Nociceptive pain	1.82	-2.37, 6.01	0.390
Maximum pain intensity	1.81	0.86, 2.76	< 0.001
Pain frequency	0.22	0.04, 0.39	0.015

Among participants experiencing chronic pain, the most common management approaches were consultation of healthcare practitioners (40.20%) and utilization of alternative medicine (33.33%). Participants with high levels of pain interference were significantly more likely to seek care from healthcare practitioners (58.33%) compared to those with low pain interference (24.07%, p 0.001). Among analgesics classified as PIMs, NSAIDs and skeletal muscle relaxants were the most frequently prescribed medications (11.88%). Anticonvulsants were prescribed significantly more often to participants with high pain interference as shown in Table 4.

**Table 4:** Pain management and evidence of PIMs use among participants with chronic pain categorized by perceived interference

Factors	Total *N* = 102	Interference		*p*-value
		Low *N* = 54	High *N* = 48	
Most common strategy for pain management, *n* (%)				0.001
Enduring/Distraction	26(25.49)	21(38.89)	5(10.42)	
Self-seeking oral painkillers	1(0.98)	0(0)	1(10.42)	
Practitioners	41(40.20)	13(24.07)	28(58.33)	
Alternative medicine	34(33.33)	20(37.04)	14(29.17)	
Potentially Inappropriate Medication use, *n* (%)				
NSAIDs	19(11.88)	4(7.41)	9(18.75)	0.136
Skeletal muscle relaxant	19(11.88)	6(11.11)	8(16.67)	0.566
Anticonvulsant	7(4.38)	0(0)	7(14.58)	0.004
Opioids	5(3.12)	1(1.85)	4(8.33)	0.185
Paracetamol	10(6.25)	4(7.14)	6(12.50)	0.510
Topical drug	62(38.75)	26(48.15)	29(60.42)	0.238
Alternative medicine, *n* (%)				
Massage	26(16.25)	12(22.22)	14(29.17)	0.497
Compression	22(13.75)	14(25.93)	8(16.67)	0.336
Cupping	1(0.62)	0(0)	1(2.08)	0.471
Needling	2(1.25)	0(0)	2(4.17)	0.219
Exercise	12(8.12)	6(11.11)	7(14.58)	0.768

## Discussion

In this study, while only about two-thirds of the study population reported currently experiencing chronic pain, nearly 90% had a history of chronic pain at some point in their lives. Notably, half of those with current chronic pain had not received a formal assessment or diagnosis. Pain interference was highest in respect to mobility and daily activities. Age, maximum pain severity, and pain frequency were significantly associated with pain interference. In the group of patients reporting high-interference participants were being more likely to seek professional care. NSAIDs and skeletal muscle relaxants were the most frequently utilized PIMs, while anticonvulsants were more commonly used in those with high pain interference. This finding highlights the often-overlooked presence of chronic pain among older adults who regularly attend our outpatient clinic for chronic disease management. Pain interference significantly affects their quality of life, particularly in activities of daily living (ADL). These results underscore the importance of incorporating routine pain assessment and individualized pain management strategies into standard care within this setting.

### Prevalence of chronic pain and its underestimation

Older adults with chronic diseases are more likely to experience chronic pain. The prevalence observed in this study is in alignment with some prior studies conducted in China, Sweden, and Finland [18, 31, 32] but is higher in comparison to studies involving participants with a lower age range and absence of chronic diseases [13, 33, 34]. A significant association exists between the presence of chronic pain and chronic diseases, particularly regarding the incidence of multiple chronic diseases [13, 34]. This study emphasizes the impact of chronic disease burden on chronic pain and reflects differences in subject demographics, age inclusion, healthcare system characteristics, survey method or the definition of chronic pain used. The study may be specific to Thailand and its culture. Therefore, the results have to be interpreted in such light [35].

Our study reveals that, despite the relatively high prevalence of chronic pain among older adults with chronic diseases, compared to prevalence rates reported in other studies conducted in the general older adult population, which range from 40.3% to 57.3% [13, 18, 31]”.

This statement has also been repositioned and clearly identified as part of the discussion of our findings. However, it should be noted that some of these studies included participants with a higher age range, such as those over 65 years old, or used different scales for pain measurement, such as the Visual Analogue Scale (VAS) [31]. Approximately 50% of participants with current chronic pain had not been assessed or diagnosed by a physician, which may reflect challenges in pain diagnosis, assessment, or documentation in clinical practice. Consistent with previous studies, this research underscores insufficient recognition of chronic pain by health care practitioners in primary care settings [27, 36–38]. The underestimation of chronic pain assessment can occur for various reasons. In addition to specific patient conditions that may hinder self-reporting of pain, such as cognitive impairment [13], discordance in information exchange and doctor–patient interaction can also contribute to underestimation in some settings. Therefore, empathetic listening and patient-centered medicine approach (PCM) from healthcare practitioners are essential to address these challenges [39]. Furthermore, factors beyond our study setting include older patients’ perceptions and coping strategies regarding their pain. Some view pain as an inevitable part of aging, do not expect improvement, or choose to self-manage their pain despite experiencing varying degrees of pain severity [40]. Further studies are needed to explore the underlying factors contributing to these findings, which could help inform more effective strategies for improving care for chronic pain.

### Perceived interference and associated factors

Chronic pain has generally been found to interfere primarily with mobility and daily activities, findings supported by this study, regardless of the various measurement tools used [13, 18, 34, 41–43]. This highlights the significant impact of chronic pain on various life dimensions, significantly impairing physical functioning activity and functional ability, underscoring the profound impact of chronic pain on the quality of life [15]. However, there is limited evidence examining the association between chronic pain interference and other factors. This study identifies a significant positive correlation between pain interference and the level of pain severity, pain frequency, and increased patient age, findings also supported by prior studies conducted in older adults with musculoskeletal pain [43–45]. Some theoretical explanations exist about age-related changes in pain processing. One explanation is that aging often leads to reduced pain adaptability and increased pain sensitivity [46]. Another study suggests that aging is associated with a decrease in pain threshold and results in an increase in the prevalence of pain complaints [47]. Consequently, older adults reporting high pain interference scores tend to experience greater disruption in key aspects of daily life, as reflected in BPI domains such as sleep, work, and mood, which may collectively contribute to reduced nutritional intake and overall quality of life [13, 18, 34, 35, 43, 48]. In combination with the current issue of pain underestimation, these findings stress the importance of proper chronic pain management in older adults i.e. consideration of appropriate pain modalities that address both pain severity and pain duration. A previous study reveals that pain interference significantly influences the physical performance of the patient beyond the impact of pain intensity alone [48]. Therefore, the proper assessment of pain interference and adequate pain management, which consider both duration and intensity, should be prioritized when caring for older adults with chronic pain and chronic diseases [43, 48–50].

### Pain management strategies and the use of medication

Utilizing alternative medicine provided by professionals (33.33%) was common among participants experiencing chronic pain with lower interference. In addition, self-applying topical medication was a common method in both interference groups. Similar trends of adopted alternative medicine strategies such as massage were also highlighted in the study from China [18]. These findings reflect the broad acceptance of culturally relevant practices in different areas in Asia. A previous study reported that massage therapy is considered as a means of improving emotional health, resulting in more energy, less fatigue, better social functioning, and even better overall health in older adults experiencing persistent pain [51]. However, the safety and efficacy of alternative approaches should be thoroughly assessed to ensure implementations are evidence-based. Interestingly, many older adults in our study tended to endure pain, but this strategy was less frequently reported among those with severe interference. Psychological management, such as cognitive-behavioral therapy or mindfulness, is an established component of pain care internationally [52–54]. In Thailand, however, these approaches are not widely available in routine practice for older adults and are more commonly applied in the cancer care context [55]. Further studies assessing the role and feasibility of psychological strategies in pain management would be useful.

Higher pain interference increases the possibility of seeking help from medical practitioners, while individuals with lower pain interference may have more low-risk ways to deal with the pain such as alternative medicine or self-management. Thus, the provision of education for older adults with chronic diseases and pain on initial-self management strategies may improve under appreciation of chronic pain issues. Apart from oral analgesics classified as potentially inappropriate medications (PIMs) that were observed to be used for managing chronic pain, paracetamol (6.25%) was also reported, which is commonly used as a first-line therapy. Additionally, topical medications were reported for both low and high levels of pain interference [56]. Commonly prescribed drugs like NSAIDs and skeletal muscle relaxants, despite their higher risks in older adults, may sometimes be justified for more severe pain under careful monitoring in a healthcare setting [57]. Weak opioids including tramadol and codeine, along with anticonvulsants such as gabapentin and pregabalin, were primarily prescribed for participants with severe pain interference in this study. Evidence from several studies suggests that opioids provide benefits in older adults with chronic pain and multimorbidity, particularly improving physical functioning. However, most studies focus on short-term use (seven days to six weeks), emphasizing suitability of opioids for acute pain, while cautioning about side effects [58–60]. Gabapentinoids, commonly used for chronic pain particularly in neuropathic pain, reduce pain sensitivity by modulating afferent excitability of neurons. In older adults, they should be started at a low dose and monitored for adverse effects [61].

Although oral analgesics classified as PIMs were commonly observed in this study, most were prescribed following consultation with a physician. It is important to note that “potentially” does not mean “definitely inappropriate [26]”. However, due to limitations in documentation, our study cannot determine the true appropriateness of these prescriptions. When continuing to prescribe medications categorized as PIMs, physicians should carefully minimize risks for older adults by considering indications and contraindications, weighing risks and benefits, managing comorbidities, and deprescribing as soon as symptoms resolve [62]. As supported by our study, non-pharmacological management may play an increasingly important role in the treatment of pain in older adults (60). Our findings indicate that participants with chronic pain use topical drug, as well as professionally administered alternative therapies such as massage, compression, cupping, and needling. Moreover, the use of these pain management strategies tends to increase as pain interference becomes more significant [63]. Interestingly, while our study found that self-medication with painkillers was reported by only one participant, which is notably lower than the proportions reported in a systematic review by Hughes et al. [64], this discrepancy may be partly explained by our data collection method, which asked participants to select only one best choice representing their most commonly used pain management strategy. Differences in culture and accessibility of pain medications may also play a role.

### Strengths and limitations of the study

This study not only reports the prevalence, characteristics and management strategies of chronic pain among older adults with chronic diseases in a primary care unit in Thailand, but also examines the impact of chronic pain and its associated factors. By addressing a more comprehensive understanding of its multidimensional effects of chronic pain within the healthcare system. However, several limitations should be noted. First, as a cross-sectional study, the ability to infer causal relationships between pain descriptors and interference is limited; the design only provides a snapshot of pain experiences and related factors, rather than tracking changes over time as in a longitudinal studies. Second, due to specific features of the healthcare system in Thailand that may influence medication choices and management strategies, these findings may have limited applicability to other regions or to settings with different cultural attitudes toward chronic pain. Furthermore, as data were collected from a single university-affiliated hospital, the findings may not be fully generalizable to community-dwelling older adults in Thailand or to other healthcare facilities with different practice patterns. Access to care issues – for example, health scheme coverage or geographic location – may influence the availability and use of pain control. Data from populations with different backgrounds may therefore yield different results. Finally, the study did not explore patients’ perceptions or coping strategies in depth, which might be better captured through qualitative research approaches.

## Conclusion

This study highlights the high prevalence of chronic pain, affecting more than 60% of older adult patients with chronic diseases. Our findings highlight several new insights that are particularly relevant to the Thai healthcare context. First, older adults with high pain interference experienced distinctly more severe pain and demonstrated different help-seeking behaviors compared to those with low interference. Second, mobility limitation emerged as a chief complaint associated with chronic pain. Third, many older adults tended to endure pain without medication until symptoms became severe. Finally, current pain management reflected a mixture of conventional care, alternative therapies, and, in some cases, the use of potentially risky medications.

Collectively, these findings reinforce the need for comprehensive and tailored pain assessment and management strategies for older adults, with the goal of reducing pain intensity and frequency and thereby minimizing its interference with daily life. The provision of education to both physicians and patients on appropriate chronic pain assessment, management strategies and options may benefit older adults with chronic diseases. In clinical practice, comprehensive pain management strategies including PIMs use and support for non-pharmacological treatments are also crucial in clinical practice. Future policies should prioritize pain management in aging populations, ensuring that treatment plans are both effective and safe while addressing the specific needs of older adult patients. Further research is needed to examine clinical decision-making and pain evaluation practices in routine care to enhance the quality and safety of pain management in older adults.

## Data Availability

The datasets used and/or analyzed during the current study are available from the corresponding author on reasonable request (contact KP).

## Abbreviations

BPI: Brief Pain Inventory
EMR: Electronic medical record
NCDs: Non-communicable diseases
NSAIDs: Non-steroidal anti-inflammatory drugs
OPD: Outpatient clinic
PCM: Patient-centered medicine approach
PIMs: Potentially inappropriate medications
